# miR-34a induces cellular senescence via modulation of telomerase activity in human hepatocellular carcinoma by targeting FoxM1/c-Myc pathway

**DOI:** 10.18632/oncotarget.2905

**Published:** 2015-01-09

**Authors:** Xinsen Xu, Wei Chen, Runchen Miao, Yanyan Zhou, Zhixin Wang, Lingqiang Zhang, Yong Wan, Yafeng Dong, Kai Qu, Chang Liu

**Affiliations:** ^1^ Department of Hepatobiliary Surgery, The First Affiliated Hospital of Xi'an Jiaotong University, Xi'an 710061, China; ^2^ Department of Obstetrics and Gynecology, University of Kansas School of Medicine, Kansas City, KS 66160, USA

**Keywords:** miR-34a, HCC, senescence, telomerase, telomere

## Abstract

Increasing evidence suggests that miRNAs can act as either tumor suppressors or oncogenes in carcinogenesis. In the present study, we identified the role of miR-34a in regulating telomerase activity, with subsequent effect on cellular senescence and viability. We found the higher expression of miR-34a was significantly correlated with the advanced clinicopathologic parameters in hepatocellular carcinoma. Furthermore, tumor tissues of 75 HCC patients demonstrated an inverse correlation between the miR-34a level and telomere indices (telomere length and telomerase activity). Transient introduction of miR-34a into HCC cell lines inhibited the telomerase activity and telomere length, which induced senescence-like phenotypes and affected cellular viability. We discovered that miR-34a potently targeted c-Myc and FoxM1, both of which were involved in the activation of telomerase reverse transcriptase (hTERT) transcription, essential for the sustaining activity of telomerase to avoid senescence. Taken together, our results demonstrate that miR-34a functions as a potent tumor suppressor through the modulation of telomere pathway in cellular senescence.

## INTRODUCTION

Hepatocellular carcinoma (HCC), the second leading cause of cancer-related deaths worldwide, is rarely detected early and is usually fatal within the months after diagnosis [1]. Despite the extensive investigation of the disease in the past years, its molecular mechanism still remains elusive [2]. Recent studies indicate that cancer cell senescence, a state of irreversible growth arrest, is a potential mechanism of HCC regression following chemotherapy [3]. It was found that TGF-*β*-induced senescence was associated with strong treatment response against HCC [4]. Additionally, our previous results also demonstrated that cellular senescence would contribute to the chemotherapy response among HCC patients [5, 6].

Cellular senescence can be triggered by different mechanisms including telomere shortening, epigenetic derepression of INK4a/ARF locus, and DNA damage [7]. In liver, hepatocytes exhibiting telomere dysfunction would undergo senescence, as significant barriers to cancer formation. It was reported that hepatocyte telomere shortening was accelerated in patients with chronic liver disease, accompanied with increased number of *β*-galactosidase (SA-*β*-Gal)-positive cells, a marker of senescence [8]. On the contrary, reactivation of telomerase, which is critical for maintenance of telomere length, is one of important features in most human cancers [9]. Telomerase expression would greatly enhance the transformation of liver cells, while telomerase deletion limits the progression of p53-mutant HCCs with short telomeres [10, 11]. Thus, telomere and telomerase-based therapy to induce cellular senescence in cancer was supposed to be a promising anti-tumor method [12].

miRNA consists of 22 nucleotides and regulates gene expression in a post-transcriptional manner by pairing with complementary nucleotide sequences in the 3′ untranslated regions (UTRs) of target mRNAs [13]. Since miRNAs regulate a variety of genes pivotal for senescence and apoptosis, they can function as tumor suppressors or oncogenes, depending on the involvement of specific target genes in tumorigenesis [14]. In this respect, the miRNAs targeting telomerase directly or indirectly, might serve as tumor suppressive genes in cellular processes, such as let-7g, miR-138, miR-1207 and miR-1266, which induced tumor senescence and death eventually [15–17]. Recently, the miR-34 family (a, b and c) has gained attention as they were identified as p53 targets and regulate p53-mediated cycle arrest and apoptosis [18]. The miR-34 family is frequently downregulated in cancer partly due to the inactivation of p53 [19]. Previous reports showed that miR-34-induced senescence in cancer cell is all in the form of telomere-independent cell cycle arrest. Until recently, it was unknown whether miR-34 family could induce cellular senescence in HCC in a telomere-dependent way.

In the present study, we provided evidence that miR-34a, the novel prognostic marker in cancer, regulated senescence and viability in HCC tissues and cell lines, at least, by targeting the FoxM1 and c-Myc gene, in the telomere pathway.

## RESULTS

### miR-34 family expression is associated with malignant characteristics in patients with HCC

**Figure 1:**
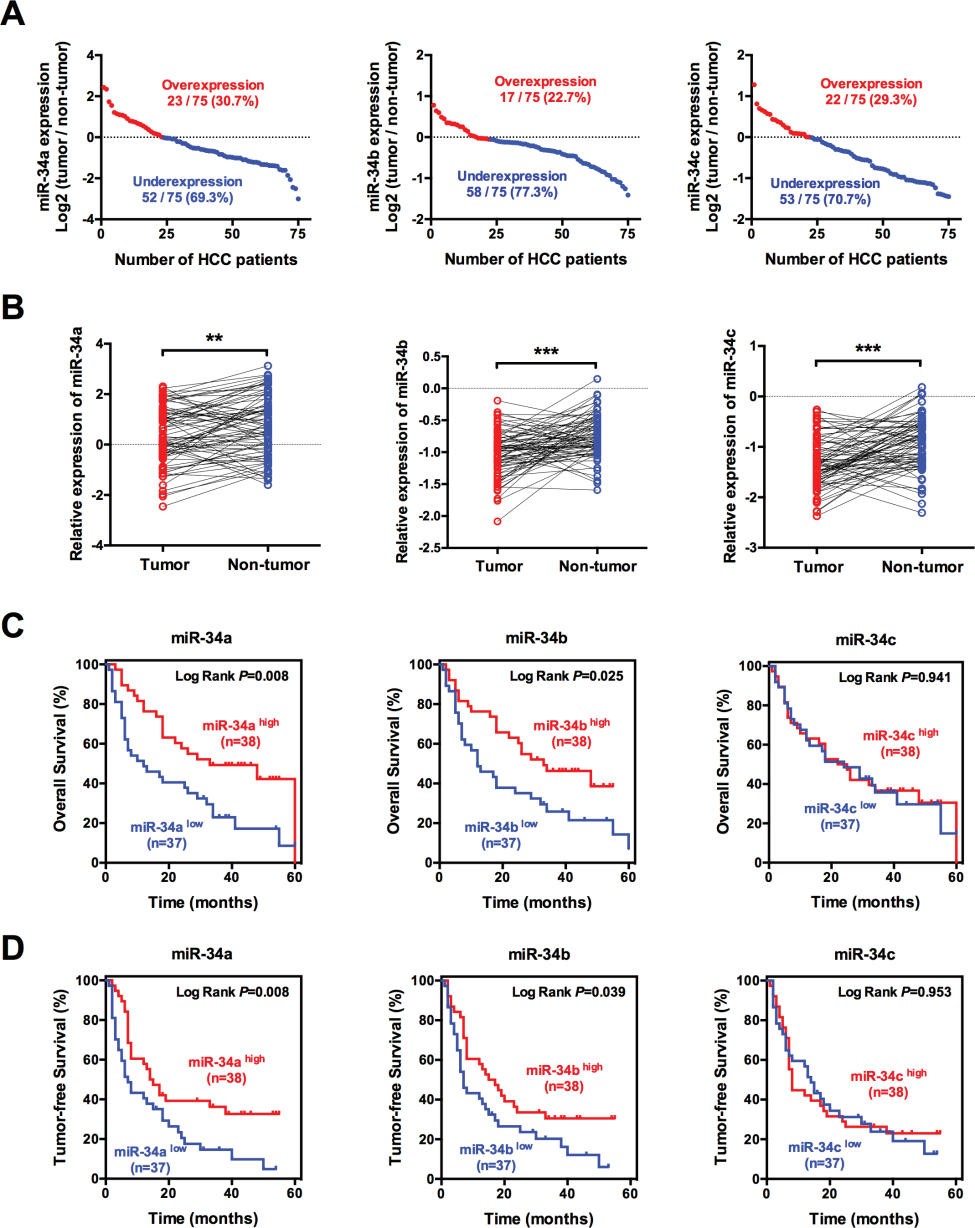
miR-34 family is frequently down-regulated in HCC and associates with poor prognosis **(A, B)** miR-34a, miR-34b and miR-34c expression were significantly decreased in HCC compared with the corresponding adjacent tissues using qRT-PCR analyses. Expression was shown as a log_2_-fold change. **(C, D)** Decrease in miR-34a and miR-34b levels were significantly correlated with the overall survival and tumor-free survival of HCC patients, whereas no substantial difference was observed for miR-34c. *P < 0.05, **P < 0.01 and ***P < 0.001.

To gain insight into the biological role of miR-34 family in human HCC development, we examined the expression levels of miR-34 family in 75 paired HCC samples by qRT-PCR. Our data showed that miR-34a, miR-34b and miR-34c were underexpressed in more than half HCC samples compared with the adjacent tissues, suggesting that down-regulation of miR-34 famlily might be involved in the hepatic carcinogenesis (*P* < 0.05, Figure 1A–1B).

To further explore the potential roles of miR-34 family in affecting malignant characteristics, the expression levels of miR-34 family in tumor tissues were used to build a signature of prognosis. For each miRNA analysis, the patients were classified as the higher miRNA expression group or the lower group by the median value as the cutoff point. Kaplan–Meier curves showed that patients with underexpressed miR-34a and miR-34b had poorer overall survival and higher recurrence rates than those with higher expression (*P* < 0.05), whereas no substantial difference was observed for miR-34c (*P* > 0.05, Figure 1C–1D). In addition, this relationship was also verified by the multivariate Cox regression analysis, which demonstrates that both miR-34a and miR-34b could be independent prognostic factors for the overall survival and recurrence in HCC patients underwent surgical resection (Table 1).

**Table 1:** Distribution of patients' characteristics and prognosis analysis

Variable	Total, *N* = 75, No. (%)	Death, *n* = 51, No. (%)	Crude HR (95% CI)	Adjusted HR (95% CI)	Recurrence, *n* = 58, No. (%)	Crude HR (95% CI)	Adjusted HR, (95% CI)
miR-34a level							
Low	37(49.3)	30(58.8)	Ref	Ref	33(56.9)	Ref	Ref
High	38(50.7)	21(41.2)	**0.48(0.28–0.85)**	**0.51(0.28–0.96)**	25(43.1)	**0.52(0.31–0.87)**	**0.51(0.29–0.91)**
miR-34b level							
Low	37(49.3)	30(58.8)	Ref	Ref	32(55.2)	Ref	Ref
High	38(50.7)	21(41.2)	**0.53(0.30–0.94)**	0.54(0.29–1.02)	26(44.8)	**0.59(0.35–0.99)**	**0.52(0.29–0.93)**
miR-34c level							
Low	37(49.3)	25(49.0)	Ref	Ref	29(50.0)	Ref	Ref
High	38(50.7)	26(51.0)	0.98(0.57–1.70)	1.34(0.73–2.74)	29(50.0)	0.99(0.59–1.65)	1.22(0.71–2.09)
Age							
< 50 years	32(42.7)	17(33.3)	Ref	Ref	22(37.9)	Ref	Ref
≥ 50 years	43(57.3)	34(66.7)	**2.17(1.20–3.92)**	**2.85(1.50–5.43)**	36(62.1)	1.5(0.96–2.82)	**1.83(1.04–3.21)**
Sex							
Female	20(26.7)	16(31.4)	Ref	Ref	17(29.3)	Ref	Ref
Male	55(73.3)	35(68.6)	0.63(0.35–1.15)	0.65(0.35–1.21)	41(70.7)	0.72(0.41–1.26)	0.66(0.37–1.19)
Cirrhosis							
No	34(45.3)	23(45.1)	Ref	Ref	27(46.6)	Ref	Ref
Yes	41(54.7)	28(54.9)	1.27(0.72–2.23)	1.25(0.67–2.34)	31(53.4)	1.10(0.66–1.85)	1.11(0.64–1.93)
Smoking							
No	43(57.3)	32(62.7)	Ref	Ref	36(62.1)	Ref	Ref
Yes	32(42.7)	19(37.3)	0.71(0.40–1.25)	0.57(0.31–1.05)	22(37.9)	0.77(0.40–1.15)	0.70(0.40–1.22)
Drinking							
No	55(73.3)	39(76.5)	Ref	Ref	44(75.9)	Ref	Ref
Yes	20(26.7)	12(23.5)	0.74(0.39–1.41)	0.68(0.34–1.35)	14(24.1)	0.72(0.40–1.32)	0.82(0.44–1.53)
ALT							
≤ 40 U/L	39(52.0)	26(51.0)	Ref	Ref	29(50.0)	Ref	Ref
> 40 U/L	36(48.0)	25(49.0)	1.05(0.60–1.84)	1.27(0.71–2.25)	29(50.0)	1.26(0.75–2.12)	**2.10(1.20–3.67)**
AST							
≤ 40 U/L	41(54.7)	24(47.1)	Ref	Ref	29(50.0)	Ref	Ref
> 40 U/L	34(45.3)	27(52.9)	1.62(0.94–2.82)	**2.18(1.23–3.86)**	29(50.0)	1.64(0.98–2.76)	**1.86(1.10–3.16)**
HBV							
Negative	18(24.0)	12(23.5)	Ref	Ref	13(22.4)	Ref	Ref
Positive	57(76.0)	39(76.5)	1.06(0.55–2.03)	1.11(0.54–2.30)	45(77.6)	1.35(0.72–2.51)	0.94(0.49–1.80)
HCV							
Negative	70(93.3)	49(96.1)	Ref	Ref	54(93.1)	Ref	Ref
Positive	5(6.7)	2(3.9)	0.34(0.08–1.43)	**0.19(0.04–0.91)**	4(6.9)	0.69(0.25–1.92)	0.50(0.18–1.42)
AFP							
< 50 ng/mL	28(37.3)	20(39.2)	Ref	Ref	21(36.2)	Ref	Ref
≥ 50 ng/mL	47(62.7)	31(60.8)	1.04(0.59–1.83)	1.01(0.54–1.90)	37(63.8)	1.18(0.69–2.01)	0.97(0.55–1.71)
Tumor size							
< 5 cm	28(37.3)	15(29.4)	Ref	Ref	18(31.0)	Ref	Ref
≥ 5 cm	47(62.7)	36(70.6)	**3.19(1.67–6.08)**	**5.22(2.56–10.64)**	40(69.0)	**2.65(1.50–4.67)**	**3.68(2.02–6.71)**
Tumor number							
Single	64(85.3)	43(84.3)	Ref	Ref	47(81.0)	Ref	Ref
Multiple	11(14.7)	8(15.7)	1.14(0.53–2.44)	0.94(0.43–2.04)	11(19.0)	1.66(0.85–3.23)	1.30(0.65–2.60)
Tumor capsule							
No	12(16.0)	8(15.7)	0.94(0.44–2.01)	0.88(0.39–2.03)	8(13.8)	0.73(0.35–1.56)	0.91(0.42–1.95)
Yes	63(84.0)	43(84.3)	Ref	Ref	50(86.2)	Ref	Ref
Tumor thrombosis							
No	62(82.7)	41(80.4)	Ref	Ref	47(81.0)	Ref	Ref
Yes	13(17.3)	10(19.6)	1.42(0.71–2.85)	1.34(0.66–2.70)	11(19.0)	1.39(0.72–2.68)	1.17(0.58–2.35)
TNM staging							
I+II	29(38.7)	22(43.1)	Ref	Ref	24(41.4)	Ref	Ref
III+IV	46(61.3)	29(56.9)	0.98(0.56–1.71)	0.66(0.37–1.19)	34(58.6)	1.09(0.64–1.84)	0.82(0.46–1.44)

In the correlation analysis, the miR-34a level was showed to be negatively correlated with AFP level (*r* = –0.236, *P* < 0.05), suggesting some connections of these markers in the prognosis of HCC patients. However, the correlations between miR-34a and other tumor index such as tumor size (*r* = –0.05, *P* > 0.05) and TNM staging (*r* = –0.094, *P* > 0.05) fail to reach statistical significance.

### Correlation of miR-34 family levels with telomerase activity

**Figure 2:**
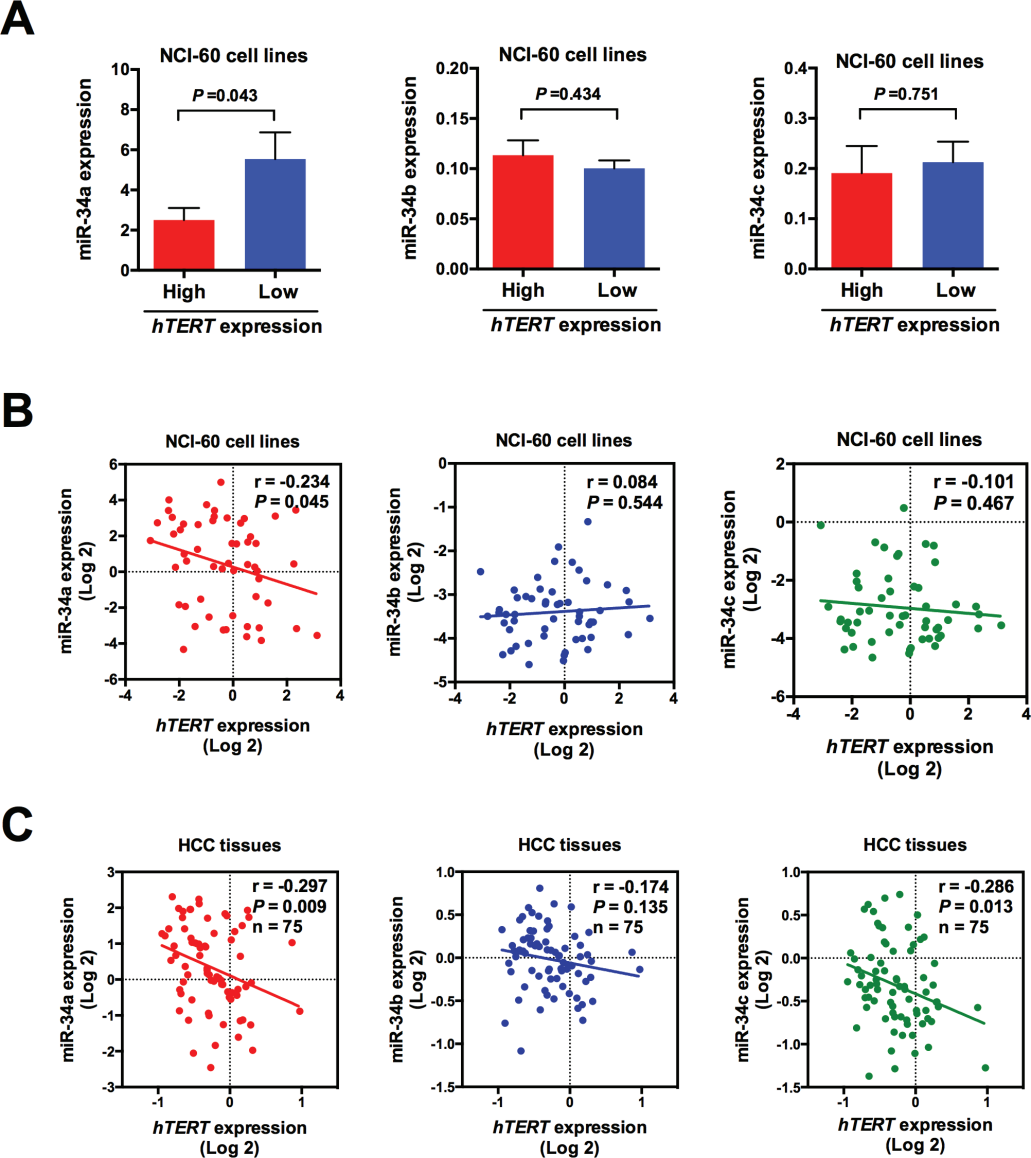
Correlation of miR-34 family levels with telomerase activity **(A, B)** Relationship between miR-34 family levels and hTERT mRNA expression in NCI-60 cell lines. The mRNA expression data derived from NCI-60 were obtained from public data. **(C)** Relationship between miR-34 family levels and hTERT mRNA expression in 75 HCC samples using linear regression models.

Previous studies demonstrated that miR-34 family could induce cellular senescence by participating in cell cycle arrest. However, they were mainly focused on the p53/p21 or p16/Rb senescence pathway, and little is known about the telomere mechanism. Thus, by computing the correlation coefficient using the NCI-60 expression profiling data, we quantified the correlation strength between miR-34 familiy and telomerase reverse transcriptase (hTERT) expression profile in different cancer cell lines (except neurologic cancer cells). As shown in Figure 2A–2B, only miR-34a is inversely correlated with the telomerase activity (*P* < 0.05), indicating the potential regulating roles of miR-34a in telomerase activity. We then examined the relationship between miR-34 family and telomerase activity in 75 HCC samples by qRT-PCR. As shown in Figure 2C, the hTERT mRNA expression appears to be inversely correlated with the levels of miR-34a (*P* < 0.05), which is consistent with the NCI-60 data.

### miR-34a inhibits telomerase activity and induces telomere shortening

**Figure 3:**
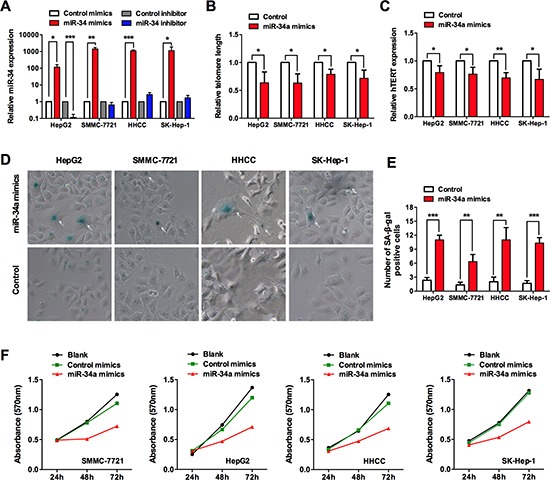
miR-34a induces senescence-associated growth arrest in liver cancer cells **(A)** After 48 h of miR-34a mimics or inhibitor transfection, miR-34a level was analyzed by qRT-PCR. **(B)** Quantification by qRT-PCR of miR-34a effects on telomerase activity, as reflected by hTERT expression. **(C)** Quantification by qRT-PCR of miR-34a effects on telomere length, as reflected by T/S ratio. **(D)** Induction of senescence-like appearance in cells by transfection of miR-34a, as subjected to SA-*β*-gal staining. **(E)** Cells with positive SA-*β*-gal staining were counted from 10 different visual fields. **(F)** Transfection of miR-34a in cancer cells inhibited cellular viability as revealed by MTT assay. *P < 0.05, **P < 0.01 and ***P < 0.001.

Subsequently, we tested whether miR-34a had an effect on telomerase activity and telomere length *in vitro*. Of different liver cancer cell lines, HepG2, SMMC-7721, HHCC, and SK-Hep-1 cells were transiently transfected with a synthetic miR-34a duplex (miR-34a mimic), or an oligonucleotide complementary to the miR-34a sequence to block its function (anti-miR-34a), or negative control scrambled siRNA (control mimics and control inhibitor). After 48 hours, the expression level of miR-34a was significantly upregulated when transfected with synthetic miR-34a duplex (Figure 3A). Results showed that compared with negative control cells, ectopic expression of miR-34a significantly inhibited telomerase activity and induced telomere shortening, as represented by hTERT mRNA expression and relative T/S ratio (telomere to single copy gene), respectively (Figure 3B–3C).

Our finding that miR-34a regulates telomere length and telomerase expression prompted us to further investigate the pro-senescent effect of miR-34a in HCC. We observed that introduction of miR-34a into liver cancer cells caused senescence-like phenotypes, with positive staining for senescence-associated *β*-galactosidase (SA *β*-gal) and enlarged cellular size (Figure 3D–3E). The cellular senescence caused by miR-34a also resulted in remarkable inhibition of cell proliferation, as represented by the MTT assay (Figure 3F). Taken together, These data suggest that suppression of cell proliferation by miR-34a is mainly associated with the induction of senescence-like phenotypes, probably via telomerase and telomere pathway.

### miR-34a targets c-Myc and FoxM1

**Figure 4:**
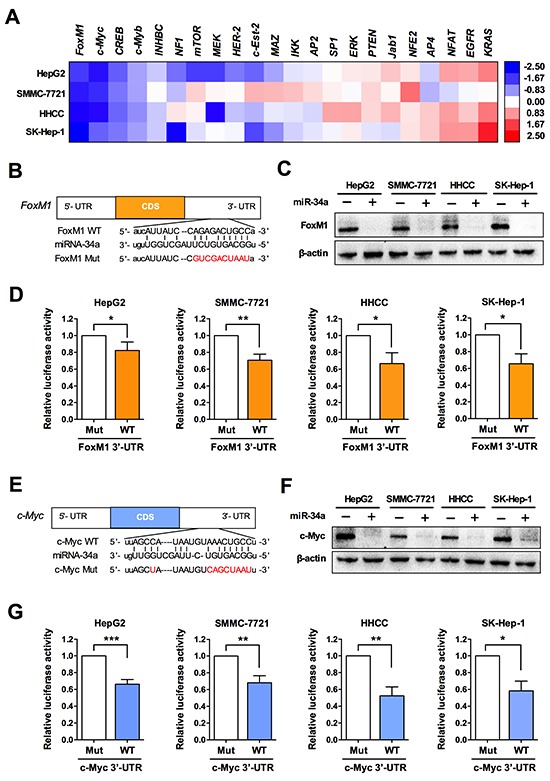
miR-34a targets FoxM1 and c-Myc in liver cancer cells **(A)** Heat maps of gene expression changes after transfected with miR-34a mimics, as revealed by qRT-PCR. The red, white, and blue right-hand panel indicates log (base 2) of expression ratios after miR-34a mimics transfection. White represents a 1:1 ratio, red indicates up-regulation, and blue indicates down-regulation. **(B, E)** miR-34a and the miR-34a-binding site in the 3′-UTR of FoxM1 and c-Myc, respectively. **(C, F)** After 48 h of miR-34a transfection, protein expression level was analyzed by western blot. **(D, G)** Luciferase assay with FoxM1 or c-Myc wild-type 3′-UTR or 3′-UTR mutated in the predicted binding site of miR-34a, transfected with pre-miR-34a. Mut, mutation; MT, wild type. *P < 0.05 and **P < 0.01.

Our above data showed that miR-34a inhibited the expression and activity of telomerase significantly. Next we sought to find out the molecular mechanism by which miR-34a regulates telomerase expression. It is known that hTERT expression can be activated by numerous transcription factors [20]. Thus we wondered whether miR-34a could affect any of the transcriptional factor expression. Mir-34a was transfected to several HCC cell lines and expression of the transcriptional factors was examined by qRT-PCR analysis. The data showed that among the hTERT activators examined, FoxM1 and c-Myc were the most down-regulated genes by miR-34a overexpression (Figure 4A). By target prediction, it turned out that FoxM1 and c-Myc have predicted binding sites for miR-34a in their 3′ UTRs (Figure 4B, 4E). With the transfection of miR-34a duplex (miR-34a) in cancer cell lines, we found that miR-34a reduced the protein levels of c-Myc and FoxM1 significantly (Figure 4C, 4F). Furthermore, to verify the idea that miR-34a represses FoxM1 and c-Myc through these sites, we generated luciferase reporter constructs, in which the miR-34a seed target or the specific 3′-UTR is placed behind the luciferase gene. Our results showed that miR-34a inhibited luciferase activity significantly, whereas no effect was observed when their respective target sites were mutated, suggesting that miR-34a directly targets c-Myc and FoxM1 via binding to the 3′ UTRs in liver cancer cells (Figure 4D, 4G).

### miR-34a inhibits telomerase activity via FoxM1/c-Myc signal pathway

**Figure 5:**
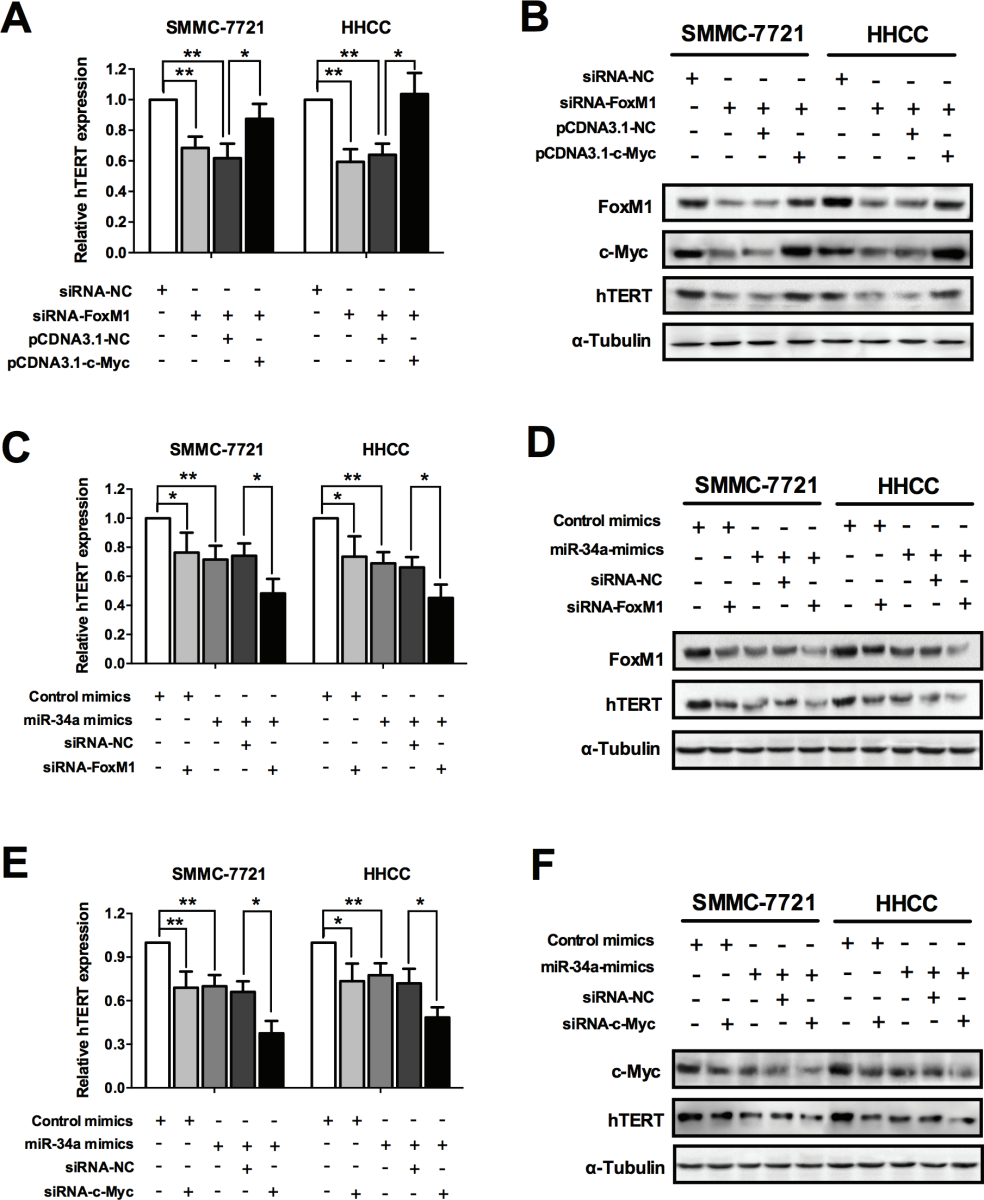
miR-34a inhibits telomerase activity via FoxM1/c-Myc signal pathway **(A, B)** The effect of c-Myc overexpression to rescue the FoxM1-mediated inhibition of hTERT, as revealed by qRT-PCR and western blot. **(C, D)** Similar effects of miR-34a mimics and siRNA-FoxM1 on the hTERT expression, with possible synergistic effect when transfected together, as revealed by qRT-PCR and western blot. **(E, F)** Similar effects of miR-34a mimics and siRNA-c-Myc on the hTERT expression, with possible synergistic effect when transfected together, as revealed by qRT-PCR and western blot. *P < 0.05 and **P < 0.01.

Since miR-34a targets FoxM1 and c-Myc, which were demonstrated to be the hTERT transactivators, we further investigated whether miR-34a inhibits telomerase activity via FoxM1/c-Myc signal pathway. It has been previously reported that FoxM1 transactivates the human c-Myc promoter via both its P1 and P2 TATA box, thus we also tried to verify this relationship in regulating telomerase activity [21]. To elucidate this mechanism, we transfected SMMC-7721 and HHCC cancer cells with FoxM1 siRNA and pCDNA3.1-c-Myc. As shown in Figure 5A–5B, the effect of c-Myc overexpression rescued the FoxM1-mediated inhibition of hTERT, as revealed by qRT-PCR and western blot, suggesting c-Myc as one of the most important downstream factors of FoxM1. As altered expression of miR-34a would contribute to the impaired telomerase activity, we discovered similar effects of miR-34a mimics and FoxM1 siRNA on the hTERT expression, with possible synergistic effect when transfected together, as revealed by qRT-PCR and western blot (Figure 5C–5D). With no surprise, similar effects were also observed for miR-34a mimics and c-Myc siRNA (Figure 5E–5F). Taken together, these results suggested that miR-34a inhibits telomerase activity significantly, probably via the FoxM1/c-Myc signal pathway.

### miR-34a induced senescence is regulated by p53

**Figure 6:**
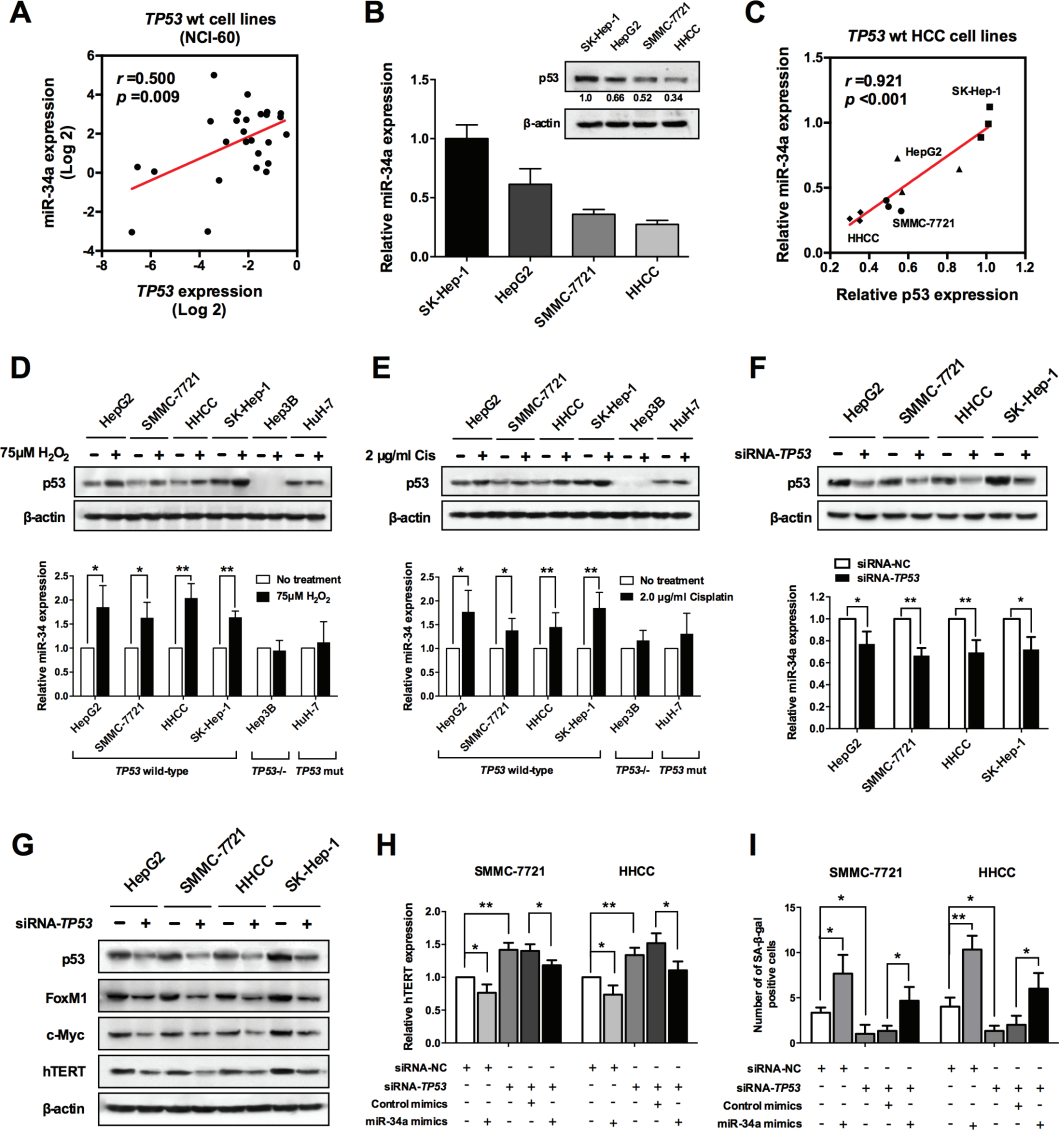
miR-34a induced senescence is regulated by p53 **(A)** Relationship between miR-34a and p53 expression levels in NCI-60 cell lines. The mRNA expression data derived from NCI-60 were obtained from public data. **(B, C)** Relationship between miR-34a and p53 expression levels in p53 wild type liver cancer cell lines. **(D–F)** Expression analysis of p53 and miR-34a level changes in different liver cancer cell lines after treatment of H_2_O_2_, cisplatin and siRNA-TP53, respectively. **(G)** After treatment of siRNA-TP53, total protein extracts were analyzed by immunoblotting, using the indicated antibodies of p53 downstream factors. **(H)** The effect of miR-34a overexpression to rescue the siRNA-TP53-mediated activation of hTERT, as revealed by qRT-PCR. **(I)** The effect of miR-34a overexpression to rescue the siRNA-TP53-mediated inhibition of cellular senescence, as revealed by SA-*β*-gal staining. *P < 0.05 and **P < 0.01.

Since previously studies reported that miR-34a is mostly activated in the presence of wild-type p53 function, we wondered whether the miR-34a induced senescence is also regulated by p53. Thus, by computing the correlation coefficient using the NCI-60 expression profiling data, we quantified the correlation strength between p53 and miR-34a expression. As shown in Figure 6A, we found a positive correlation of p53 and miR-34a in the NIC-60 data (*P* < 0.05), indicating that p53 might regulate the miR-34a function in cellular senescence. To further verify this hypothesis, we then examined the p53 and miR-34a expression in four liver cancer cells by qRT-PCR and western blot. As shown in Figure 6B–6C, the miR-34a expression positively correlated with the p53 level (*P* < 0.05), which was consistent with the NCI-60 data.

As H_2_O_2_ and cisplatin induce a senescence-like phenotype in cancer cells by activating p53, we used them to induce senescent cells in our liver cancer lines. To our expectation, the p53 protein expression significantly increased after treatment with H_2_O_2_ or cisplatin in the wild-type p53 cells and the levels of miR-34a also increased accordingly (Figure 6D–6E). However, in either the p53 mutant Huh-7 cells or the p53 deficient Hep3B cells, there is no change in either p53 or miR-34a after treatment with H_2_O_2_ or cisplatin (Figure 6D–6E). Further, when endogenous p53 expression was knocked down by a p53 siRNA in the four p53 wild-type liver cancer cells, miR-34a expression was also attenuated (Figure 6F). Subsequently, western blot also confirmed that the levels of FoxM1, c-Myc and hTERT were decreased after treatment with TP53 siRNA, indicating that the miR-34a induced senescence is probably regulated by p53 (Figure 6G). Meanwhile, results from the qRT-PCR and SA-*β*-gal staining suggested that the overexpression of miR-34a is able to rescue the siRNA-TP53-mediated hTERT activation and senescence inhibition, further demonstrating the regulatory roles of p53 in miR-34a-induced senescence (Figure 6H–6I).

### Administration of miR-34a with lentivirus induces senescence-associated growth arrest *in vivo*

**Figure 7:**
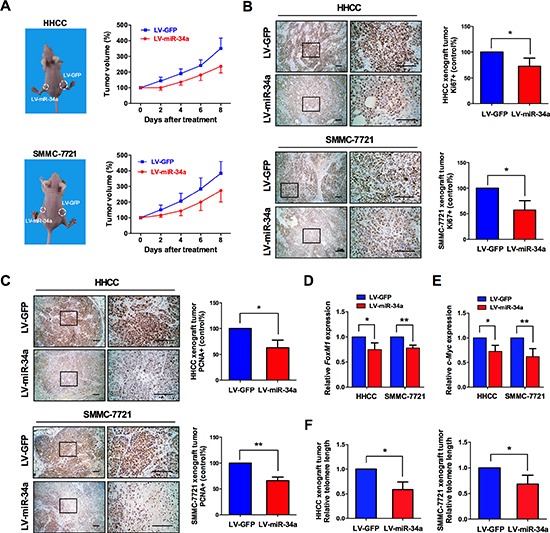
Administration of miR-34a with lentivirus suppresses cancer cell growth *in vivo* **(A)** Photographs illustrating representative features and growth curves of HHCC and 7721 tumors in nude mice after injection of LV-miR-34a or control LV-GFP. The volume was derived at day 0, when LV-miR-34a and control LV-GFP treatment was performed, and relative tumor volume was evaluated at 2-day intervals. **(B, C)** Immunohistochemistry staining for Ki-67 and PCNA in tumor tissues from mice with subcutaneous HCC implantation (left column, magnification × 40, right column, magnification × 100, scale bars 100 μm). Cells with positive staining were counted from 10 different visual fields. **(D, E)** After administration of miR-34a-expressing lentivirus, the levels of FoxM1 and c-Myc protein in the implanted tumor tissue were analyzed by western blotting. **(F)** After administration of miR-34a-expressing lentivirus, telomere length of the implanted tumor tissue was analyzed by qRT-PCR, as reflected by T/S ratio.

Subcutaneous administration of miR-34a caused significant suppression of tumor growth of both HHCC and 7721 cells (Figure 7). The average tumor volume was decreased significantly with the administration of miR-34a, compared with those administered by control miRNA (Figure 7A). In addition, tumor tissues treated with miR-34a showed a significantly decreased immunostaining of Ki67 and PCNA, suggesting the *in vivo* anti-proliferation effect of miR-34a (Figure 7B–7C). Consistent with the *in vitro* assay, we found that miR-34a significantly reduced the protein levels of c-Myc and FoxM1 in the tumors (Figure 7D–7E). In the end, telomere length was assessed in tumor tissues by qRT-PCR at 8 days after lentiviral infection. Results showed that miR-34a shortened telomere length significantly, suggesting the miR-34a-induced tumor growth arrest was probably due to the telomere-associated senescence (Figure 7F).

## DISCUSSION

In the current study, we report for the first time that miR-34a induces telomere-dependent senescence in HCC cells via targeting FoxM1/c-Myc pathway, which is regulated by p53. In addition, our data also provide *in vivo* evidences for the HCC treatment by enforced expression of the lentivirus-based miR-34a.

Recently, altered expression of microRNA genes has been reported to impact carcinogenesis [22]. Comparatively low levels of miR-34a expression were demonstrated in several types of cancers [23, 24]. However, discrepancies emerged when we focused on the miR-34a level in HCC. The expression of miR-34a was firstly reported to be down-regulated in rat during hepatocarcinogenesis induced by a methyl-deficient diet [25]. Contradictorily, other reports showd that miR-34a was also up-regulated in the HCCs in a chemical-induced HCC model [26]. Thus, we speculate that miR-34a might play varied roles during the carcinogenesis of HCCs caused by different mechanisms. In human HCCs, Li *et al*. previously reported that miR-34a was down-regulated in 19 of 25 (76%) human HCC tissues. Consistent with this result, we discovered that the expression of miR-34a was reduced in 52 of 75 (69%) human HCC tissues compared with the adjacent tissues. Furthermore, in our study, the down-regulated miR-34a level was demonstrated to be correlated with tumor malignant features and poor prognosis. Since the same chromosome 1p36 region, which is the tumor suppressor candidate, is also frequently deleted in cancer, thus the down-regulation of miR-34a could be partially due to the deletion of 1p36 [27]. However, with respect to the up-regulated miR-34a level, there might be alternative mechanisms that remain to be identified.

The association between miR-34a and senescence has been widely studied. By analyzing nutlin-3a-treated cells, Kumamoto *et al*. firstly demonstrated that miR-34 family was involved in the p53-dependent senescence pathway [28]. As well as transcriptionally regulated by p53, Christoffersen *et al*. reported that miR-34a was also regulated independently of p53 during oncogene-induced senescence [29]. In addition, they identified the c-Myc as target of miR-34a, which was consistent with our result. Recently, Bai et al. suggested that miR-34a promoted renal senescence by suppressing mitochondrial antioxidative enzymes with a concomitant increase in reactive oxygen species (ROS), providing a new mechanism of senescence pathway by miR-34a [30]. However, so far all the reported examples of senescence induction by miR-34a are telomere-independent [31, 32]. Although Jin et al. suggested that miR-34a might regulate the telomere length by targeting PNUTS, the specific mechanism still remains elusive [33]. Until recently, it was unclear whether replicative senescence could also be induced in cancer. In this study, we found that miR-34a might induce senescence in HCC via the modulation of telomerase activity, providing new insights into the mechanism underlying HCC senescence.

When focusing on the genes potentially affect telomerase activity, we identified FoxM1 and c-Myc as targets of miR-34a. c-Myc was reported to have direct roles in the induction of telomerase activity, in which it preserves chromosome integrity by maintaining telomere length [34]. Nakamura *et al*. reported that c-Myc activates telomerase by inducing expression of its catalytic subunit, TERT, as the promoter of TERT contains numerous c-Myc-binding sites that mediate transcriptional activation [35]. On the other hand, FoxM1, the upstream regulators of c-Myc, was also shown to potentially affect the telomerase activity, as TERT was found to be inhibited in the FoxM1-depleted cells [21, 36]. These evidence suggest that by targeting FoxM1/c-Myc pathway, miR-34a might possess a significant role in the regulation of telomerase activity, telomere length, and the subsequent cellular senescence.

DNA damage is one of the common pathways in the generation of senescence, whether induced by telomere dysfunction, oncogene activation, or reactive oxygen species (ROS) accumulation [37]. As a major element in the DNA damage-induced senescence, p53 inactivation was characterized in most HCC. Previous reports studying p53 and its main target p21Cip1, which were involved in the activation of cyclin-dependent kinase inhibitors (CDKIs) to induce permanent cell cycle arrest, were the main focus of extensive research leading to the recognition of senescence in cancer. However, in this study, we showed that miR-34a, which was also regulated by p53, induced telomerase activity by targeting FoxM1/c-Myc pathway, for the first time to demonstrate miR-34a as a bridge between p53 and telomere-dependent pathways in senescence.

**Figure 8:**
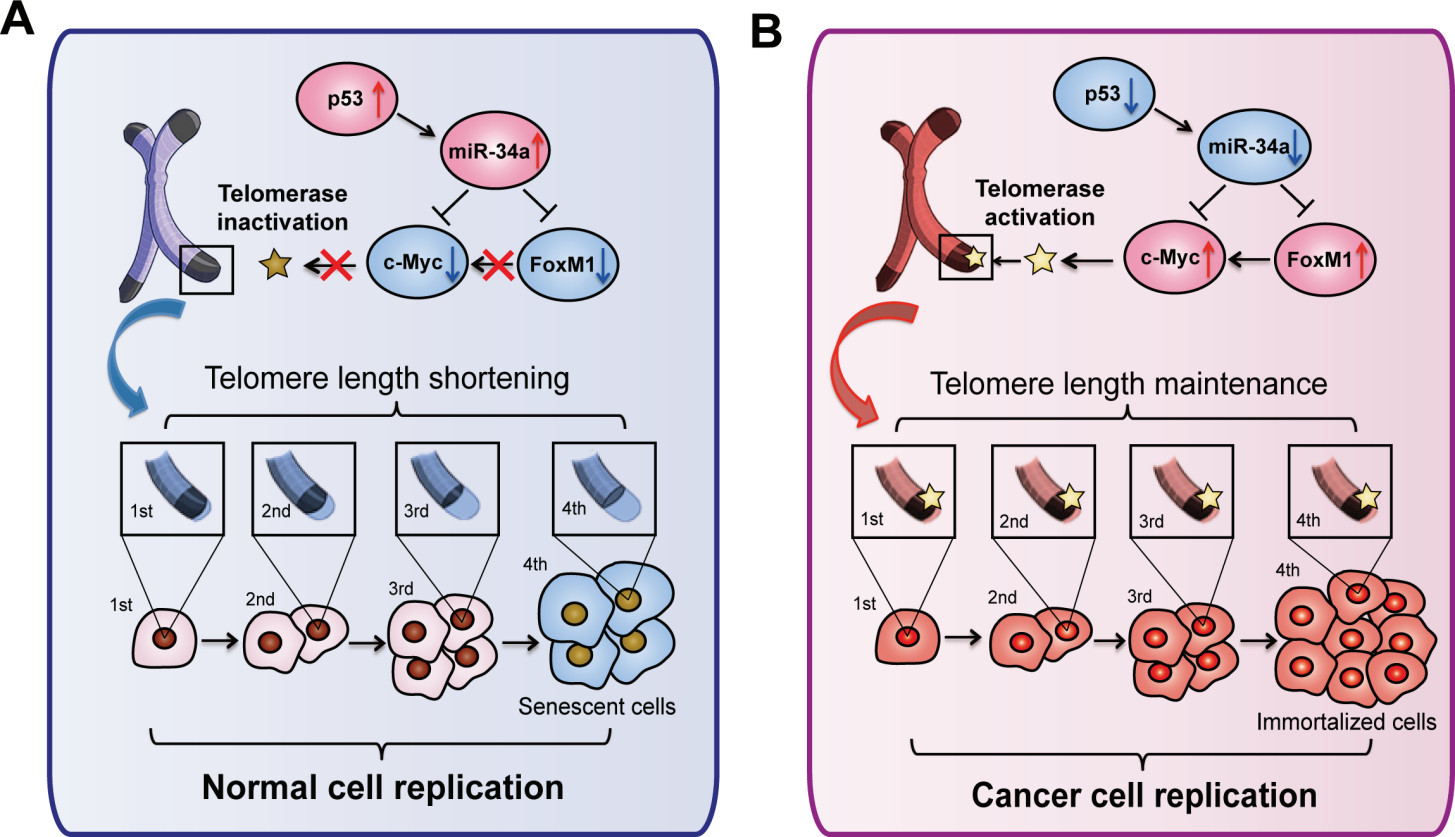
Overview of telomere related pathways for miR-34a-induced senescence In normal cells, the miR-34a activated by p53 would target FoxM1 and c-Myc expression, resulting in the inactivation of telomerase and the subsequent telomere length shortening. In contrast, in HCC cells with p53 inactivation, the suppressive miR-34a was unable to inhibit FoxM1 or c-Myc expression, leading to the sustained activation of telomerase and the subsequent telomere length maintenance.

Wierstra I *et al*. first reported that FoxM1c transactivates the human c-Myc promoter via both its P1 and P2 TATA box [21]. On the contrary, FoxM1 was also predicted bioinformatically to be the genomic target of c-Myc, and verified by chromatin immunoprecipitation assay [38]. In addition, Blanco-Bose *et al*. also reported that c-Myc binds to a conserved E-box in the promoter of FoxM1 depending on specific treatment [39]. Thus, the specific interaction between FoxM1 and c-Myc needs firther confirmation in future. In this study, we clearly demonstrate that miR-34a suppresses the expression of FoxM1 and c-Myc, which activates telomerase activity and extends telomere length, providing a rationale for the accelerated senescence by miR-34a in cancer cells (Figure 8A–8B).

In conclusion, given a critical role for senescence induction in tumor suppression and cancer treatment, our present findings have significant clinical implications. Our data demonstrate that upregulation of miR-34a would induce cellular senescence and growth arrest in human HCC. This novel miR-34a-mediated mechanism offers a new potential strategy for cancer therapy.

## MATERIALS AND METHODS

### Cell lines and human samples

Human HCC cell lines HepG2, SMMC-7721, HHCC, SK-Hep-1, Hep3B and Huh-7 were were obtained from Shanghai Institute of Biochemistry and Cell Biology, Chinese Academy of Sciences (Shanghai, China). Human HCC liver samples were collected at the time of surgical resection at the first affiliated hospital of Xi'an Jiaotong University (Xi'an, China), from 2008 to 2013. Samples were either immediately snap-frozen in liquid nitrogen and stored at –80°C or fixed in 10% formalin for paraffin embedding. Use of human tissues was approved by the Institutional Review Board of the Xi'an Jiaotong University, and written informed consent was obtained from each patient.

NCI-60 is a dataset of gene expression profiles of 60 National Cancer Institute (NCI) cancer cell lines. These 60 human cancer cell lines are derived from patients with leukaemia, melanoma, along with, lung, colon, central nervous system, ovarian, renal, breast and prostate cancers. Gene expression (GSE32474) and miRNA expression (GSE26375) data were obtained from Gene Expression Omnibus (GEO). Expression correlation between miR-34 family and hTERT or TP53 expression profile were analyzed using those two database. The p53 wild type and inconclusive cell lines was distinguished from the mutation type by the database website (p53.free.fr/Database/Cancer_cell_lines/HCC.html).

### Immunohistochemical staining

For immunohistochemical assay, slides were incubated with biotin-label goat anti-mouse or anti-rabbit IgG, followed by horseradish peroxidase (HRP) to label streptavidin. The intensity of immunohistochemical staining was scored as 0 (negative), 1 (weak), 2 (moderate strong) or 3 (strong). The extent of staining was assessed based on the percentage of positive cancer cells: 0 (negative), 1 (1–25%), 2 (26–50%), 3 (51–75%), and 4 (76–100%). The final staining score was the mean of the sum of the intensity and extent scores from three fields. The expression was considered as low if the final score was 1–5 and as high if the final score was 6–12.

### Cell culture and transient transfection

Cells were cultured in DMEM supplemented with heat-inactivated 10% FBS, 100 U/ml penicillin and 100 μg/ml streptomycin (Invitrogen, Carlsbad, CA, USA) at 37°C in 5% CO_2_. The miRNA mimics were synthesized by GenePharma (Shanghai, China). The transfection of miR-34a duplex oligoribonucleotides (sense, 5′-UGG CAG UGU CUU AGC UGG UUG UU-3′; antisense 5′-CAA CCA GCU AAG ACA CUG CGA AA-3′), mimics control (sense, 5′-UUC UCC GAA CGU GUC ACG UTT-3′; antisense, 5′-ACG UGA CAC GUU CGG AGA ATT-3′), inhibitor anti-miR-34a (5′-ACA ACC AGC UAA GAC ACU GCC A-3′), and inhibitor control (5′-CAG UAC UUU UGU GUA GUA CAA-3′) were performed using Lipofectamine 2000 (Invitrogen, Carlsbad, CA, USA) according to the procedure recommended by the manufacturer.

### Xenograft mouse model and lentivirus infection

Animal experimental protocols were approved by our institute's Committee for Ethics in Animal Experimentation. Hep3B or HHCC cells were inoculated with 5 × 10^6^ cells per site bilaterally on the backs of nude mice aged 6 weeks. Lentiviral constructs containing pre-miR-34a (LV-miR-34a) was purchased from GeneChem (Shanghai, China). As a control, we also generated a lentiviral vector that expressed green fluorescent protein alone (LV-GFP). Once the tumor size reached ~50 mm^3^, mice were treated with intratumoral injections (at days 0) of 1 × 10^7^ pfu/ml of the LV-miR-34a or LV-control lentivirus construction on each side. Tumor size was monitored by measuring the length and width with calipers, and volumes were calculated with the formula: (L × W^2^) × 0.5, where L is length and W is width of each tumor.

### Quantitative reverse transcription–polymerase chain reaction (qRT–PCR)

Total RNA was extracted from the liver samples or cultured cells using Trizol (Invitrogen, Carlsbad, CA, USA) according to the manufacturer's protocol. qRT-PCR was performed using the SYBR^®^ PrimeScript™ miRNA RT-PCR Kit and SYBR^®^ Premix Ex Taq™ (TaKaRa Biotechnology, Dalian, China). The mRNA expression was assayed in triplicate and normalized to different reference genes, such as the GAPDH, U6 (miR-34a). The relative levels were calculated using the Comparative-Ct method (^ΔΔ^Ct method). All primer pairs were synthesized by TaKaRa.

### Measurement of telomerase activity

As hTERT expression was reported to be a more sensitive marker of telomerase activity than the assessment by telomerase repeat-amplification protocol (TRAP) assay, and hTERT expression is a rate-limiting determinant of the enzymatic activity, thus we analyzed the expression of hTERT mRNA to investigate the telomerase activity. The qRT-PCR was performed as mentioned above.

### DNA extraction and measurement of telomere length

Genomic DNA was isolated from liver tissues using Tiangen DNA isolation kit (Tiangen biotech, Beijing, China). Relative mean telomere length was measured by qRT-PCR measurement of the ratio of telomere repeat units (Tel) to a single-copy gene (CON), as described previously [40]. In brief, each sample was amplified for telomeric DNA and for human β2-globulin (HBG), a single-copy control gene that provides an internal control to normalize the starting amount of DNA.

### Western blot analysis

Equal amounts of total proteins were separated and transferred to polyvinylidene difluoride membranes (Millipore, Bedford, MA, USA). The membranes were subsequently immunoblotted with the appropriate primary antibody at 4°C for 12 h, and then incubated with HRP conjugated anti-goat or anti-rabbit antibody (Santa Cruz). Signals were detected using the ECL Kit (Pierce, Rockford, IL).

### Cell proliferation assay

Cells were seeded in 96-well culture plates at a density of 5 × 10^3^ cells per well and transfected with the desired miRNA followed by incubation for 48 h. For the MTT assay (Bio Basic Inc., Canada), 20 μL of MTT solution (5 g/L) was added to each well, and the cells were incubated for 4 h. Then supernatants were removed and formazan crystals were dissolved in 200 mL dimethylsulfoxide (DMSO). After the insoluble crystals were completely dissolved, the absorbance values at 490 nm were measured using a microplate reader (Bio-Rad, Hercules, CA, USA).

### Luciferase reporter assay

Plasmids containing wild-type Luc-FoxM1, mutant Luc-FoxM1, wild-type Luc-c-Myc or mutant Luc-c-Myc 3′-UTR were specifically synthesized. Firefly luciferase and Renilla luciferase function as a tracking gene in these plasmids. Luciferase assays were performed by using the dual luciferase reporter assay system (Promega, Wisconsin, USA) 48 h after transfection, according to the manufacturer's protocol. Firefly and Renilla luciferase activities were measured by the Luc-Pair miR Luciferase Assay Kit (GeneCopoeia). Activities were normalized to Renilla luciferase.

### Senescence-associated β-galactosidase (SA β-gal) assay

Senescent cells were analyzed using a SA *β*-gal staining kit (Beyotime Inc., Nantong, China) according to the manufacturer's instructions. The percentage of SA-*β*-gal positive cells was calculated by counting the cells in 5 random fields (at least 100 cells) using bright-field microscopy, according to the method reported by te Poele et al [41].

### Clinicopathologic correlation and statistical analysis

The clinicopathologic features were analyzed as described previously [5]. All values were reported as means ± standard deviation (SD) and analyzed by SPSS 19.0 software (SPSS Inc., Chicago, IL, USA). The Fisher exact test was used for analysis of categorical data, the independent *t* test was used for continuous parametric data. *P* < 0.05 was considered statistically significant. Mean values of three independent experiments were presented for all samples.
